# Health-related quality of life among people living with type 2 diabetes: a community based cross-sectional study in rural Nepal

**DOI:** 10.1186/s12889-019-7506-6

**Published:** 2019-08-27

**Authors:** Sailendra Thapa, Prajjwal Pyakurel, Dharani Dhar Baral, Nilambar Jha

**Affiliations:** 1 Department of Public Health, Manmohan Memorial Institute of Health Sciences, Kathmandu, 44614 Nepal; 2 School of Public Health and Community Medicine, BP Koirala Institute of Health Sciences, Dharan, 56705 Nepal

**Keywords:** Quality of life, Type 2 diabetes, Rural area, D-39 questionnaire

## Abstract

**Background:**

Diabetes as being a chronic disease with a number of complications deteriorates the quality of life among the people with type 2 diabetes. Health related quality of life is widely used as an important health outcome measure worldwide. This study assessed the quality of life among the people living with type 2 diabetes in rural area of eastern Nepal.

**Methods:**

A cross sectional study was conducted among type 2 diabetic patient of rural area of eastern Nepal. Pre-tested Nepali version of D-39 questionnaire was administered through face to face interview to assess the quality of life. Door to door visit was done to identify all the type 2 diabetic patients residing in Baniyani village. Data was entered in Micro-soft excel 2007 and further processed in SPSS v.11.5 for analysis.

**Results:**

Highest quality of life mean (SD) score was in social burden domain (56.26 ± 12.07), followed by sexual functioning domain (54.35 ± 9.47), Anxiety and worry domain (54.33 ± 7.76), energy and mobility domain (51.46 ± 8.73) and diabetes control domain (50.08 ± 10.84). There was negative correlation between age and domains sexual functioning (*p* = 0.001) and energy and mobility (*p* = 0.002). In bivariate analysis, there was significance difference by sex in sexual functioning (*p* = 0.002), educational status in diabetes control (*p* = 0.021), smoking habit in energy and mobility (*p* = 0.038), duration of disease in diabetes control (*p* = 0.002) and sexual functioning (*p* = 0.001), presence of co-morbidity in social burden (*p* = 0.034) and family history of diabetes in anxiety and worry (*p* = 0.042).

**Conclusion:**

Increasing age affects sexual life and mobility of the type 2 diabetic patient. The domain sexual functioning is difference by sex and presence of co-morbidity. Similarly, domain diabetic control is affected by duration of disease and educational status of the patient. And having family history of diabetes affects the mental state of the type 2 diabetic patient.

## Background

World Health Organization (WHO) recognizes diabetes as one of the most important cause for preventable mortality and morbidity among non-communicable diseases worldwide. In 2018 there are more than 500 million people living with type 2 diabetes (T2DM) worldwide [1]. Change in lifestyle and behavioral factors have greater impact over increasing trend of diabetes worldwide [2, 3]. The estimated prevalence of T2DM in Nepal in 2015 was 8.4% [4] and in 2017 it was found to be 11.7% [5]. The burden of T2DM is increasing steadily in low income countries due to adoption of unhealthy lifestyle. T2DM is no more the disease of affluence as the prevalence of diabetes is also increasing in poorer section of community.

Diabetes being a chronic disease with number of complications deteriorates the quality of life among the people with T2DM. Quality of life is widely used as an important health outcome measure worldwide [6]. QOL refers to the physical, psychological, and social domains of health that are influenced by a person’s experiences, beliefs, expectations, and perceptions; therefore, health care providers should strive to understand the physical, emotional, and social impacts of chronic disease such as diabetes [7, 8].

Quality of life is a subjective measurement as many of its dimensions cannot be measured directly because it is related to perceived impact by the people over their life. It always try to quantify the consequences and self-perception of disease [9, 10]. Type 2 diabetic individuals are known to have lower quality of life and more depressive symptomatology than those without T2DM. It is accompanied by a marked reduction in patient’s quality of life and leads to higher disability adjusted life years than most diseases. The effects of T2DM include long term damage, dysfunction and failure of various organs. T2DM accounts for the majority (90%) of all diabetes case [11].

T2DM related complications are major causes of morbidity and mortality and have significant impact on the patient’s quality of life and productivity [12]. It is predicted that people with T2DM of Nepal have relatively reduced quality of life [11]. People with T2DM often feel challenged by their disease related features and complications and its day to day management demands. T2DM affects the health related quality of life through macro vascular complications associated non-vascular co-morbidity and also by total burden of disease [13]. Very few hospital based studies has been conducted to understand the quality of life among T2DM patient of Nepal [11, 13]. These studies revel poor health outcome largely affecting the quality of life of T2DM patients. Quality of life of T2DM patient in Nepal with less access to health care settings is still unknown. Hence, despite of having good assess to specialized health care in urban areas the T2DM patients shows lower quality of life, it shows an immense urgency of assessing in areas with less assess of health care. WHO-BREF tool is widely used tool to assess the quality of life but D-39 questionnaire, which is disease (T2DM) specific, used to assess the quality of life of T2DM patients. As D-39 is diabetes specific questionnaire to assess the quality of life, it gives more precise result about the dimension of quality of life that is mostly affected due to presence the condition. This questionnaire is widely used among Americans and Europeans [14, 15]. Assessing quality of life among T2DM patient helps to provide proper care and management of complications in clinical as well as community level. Hence this study attempts to revel the quality of life of T2DM patient at community level.

### Methodology

Community based cross Sectional study was conducted in Baniyani Village Development Committee (VDC) of Jhapa district of eastern Nepal. Door to door visit was done with the help of female community health volunteers (FCHVs) to identify diabetic patients. Those patients, who were not available during first visit, were subsequently followed for second and third times.

A pre-tested semi-structured questionnaire was used to elicit information on socio-demographic characteristics and disease profile. D-39 questionnaire was used to assess the quality of life among T2DM patient [15]. D-39 questionnaire consists of 39 items and 5 domains (Diabetes control, Anxiety and worry, Social burden, Sexual functioning and Energy and mobility). The questionnaire showed good internal consistency during pretesting with Cronbach alpha of 0.78. The questionnaire was translated into Nepali language and pretested among the 20 diabetic patients. Face and content validity was then established by consulting experts and searching the available literature. The obtained score was transferred to 0–100 scale according to previous literature [15] for the purpose of comparison. Collected data was coded and entered in Micro-soft excel 2007 and analysis was done in Statistical Package for Social Science (SPSS v.11.5). Data are presented as frequency, percentage, mean and standard deviation. Pearson’s correlation and analysis of variance (ANOVA) test were used to analyze the relation between independent factors and domains of quality of life.

All those diabetic patients who were more than 20 years and been diagnosed with diabetes with more than 6 months were included in this study. Institutional Review Committee (IRC) of B. P Koirala Institute of Health Sciences (BPKIHS), Dharan, Nepal provided Ethical Clearance. Informed written consent was taken from each study participant.

## Results

The study has identified 102 T2DM patients aged over 20 years and residing in Baniyani Village Development Committee (VDC) of Jhapa district of Eastern Nepal.

Table 1 [3] represents the socio-demographic characteristics of the respondents. Majority (58.8%) of the respondents were above 50 years of age and more than half (59.8%) of the respondents were male. About 28.4% of the participants were alcohol consumer. The jad/ rakshi (nepali home-made alcoholic drink) was the most (19.6%) consumed alcoholic drink followed by beer (12.7%) and vodka (4.9%).

**Table 1:** Socio demographic characteristics of the respondents

Characteristics	Categories	Frequency *n* = 102	Percentage (%)
Age	< 50 years	42	41.2
	≥50 years	60	58.8
Mean age = 55.23 ± 12.39 years			
Sex	Male	61	59.8
	Female	41	40.2
Ethnicity	Dalit/ Janajati	24	23.5
	Madeshi	6	5.9
	Muslim	11	10.8
	Brahmin/ Chhetry	61	59.8
Family type	Single	45	44.1
	Joint	57	55.9
Marital status	Married	100	98
	Widow	2	2
Educational status	Literate	79	77.5
	- Formal	54	68.4
	- Informal	25	31.6
	Illiterate	23	22.5
Smoking habit	Yes	19	18.6
	No	83	81.4
Alcohol consumption	Yes	29	28.4
	No	73	71.6
^a^Type of alcohol consumption (*n* = 29)	Beer	13	12.7
	Wine	1	1
	Vodka	5	4.9
	Jad/rakshi	20	19.6

^a^multiple response

Table 2 [3] shows the disease profile of the participants. Less than half (31.4%) of the respondents reported having diabetes for more than 5 years. The hypertension was the mostly (46.1%) reported co-morbidity followed by gastritis (8.8%).More than half (58.8%) of the respondents were suffering from complications due to diabetes. The respondents reported retinopathy as mostly (37.3%) reported complication.

**Table 2:** Disease profile of the respondents

Characteristics	Categories	Frequency *n* = 102	Percentage (%)
Duration of disease	< 5 years	70	68.6
	≥5 years	32	31.4
Presence of co morbidity	Yes	60	58.8
	No	42	41.2
^a^ Type of co-morbidity (*n* = 60)	Hypertension	47	46.1
	Depression	1	1
	Heart disease	1	1
	Dental caries	1	1
	Arthritis	1	1
	Asthma	3	2.9
	Hydrocele	1	1
	Skin disease	2	2
	Gastritis	9	8.8
Presence of complication	Yes	60	58.8
	No	42	41.2
^a^ Type of complications (*n* = 60)	Cardio vascular disease	3	2.9
	Neuropathy	8	7.8
	Nephropathy	2	2
	Retinopathy	38	37.3
	Foot damage	31	30.4
Family history of diabetes	Yes	41	40.2
	No	61	59.8

^a^multiple response

Table 3 represents descriptive statistics of the domains of quality of life of D-39. The cronbach’s alpha coefficient ranged from 0.63 (energy and mobility) to 0.80 (Sexual functioning). This study found the highest mean (SD) score was in social burden domain (56.26 ± 12.07), followed by sexual functioning domain (54.35 ± 9.47), Anxiety and worry domain (54.33 ± 7.76), energy and mobility domain (51.46 ± 8.73) and diabetes control domain (50.08 ± 10.84).

**Table 3:** Descriptive statistics of the D-39 domains

D-39 Domains	No. of items	Cronbach Alpha	Mean	SD	Minimum	Maximum
Diabetes control	12	0.679	50.08	10.8	27.54	83.46
Anxiety and worry	4	0.759	54.33	7.76	37.00	72.53
Social burden	5	0.736	56.26	12.07	28.34	87.00
Sexual functioning	3	0.801	54.35	9.47	33.67	75.87
Energy and mobility	15	0.635	51.46	8.7	32.00	85.56
Total score	39	0.762	53.49	11.2	35.76	76.48

Table 4 shows association between independent variables and domains of quality of life. Domains sexual functioning (*p* = 0.001) and energy and mobility (*p* = 0.002) were negatively correlated with age. This study found significance difference by sex in sexual functioning (*p* = 0.002), educational status in diabetes control (*p* = 0.021), smoking habit in energy and mobility (*p* = 0.038), duration of disease in diabetes control (*p* = 0.002) and sexual functioning (*p* = 0.001), presence of co-morbidity in social burden (*p* = 0.034) and family history of diabetes in anxiety and worry (*p* = 0.042).

**Table 4:** Bivariate association between independent variables and quality of life domains

Characteristics	Category	Diabetes control [Mean (SD)]	Anxiety and worry [Mean (SD)]	Social burden [Mean (SD)]	Sexual functioning [Mean (SD)]	Energy and mobility [Mean (SD)]	Overall QoL [Mean (SD)]
Age^a^		0.170	0.063	0.063	- 0.422	- 0.306	0.170
*P*-value		0.874	0.532	0.388	**0.001**	**0.002**	0.073
Sex	Male	52.08 (11.26)	51.21 (12.69)	50.04 (9.26)	52.46 (11.42)	54.36 (13.02)	52.53 (10.33)
	Female	50.40 (10.61)	52.63 (11.53)	53.54 (10.05)	51.11 (9.44)	50.42 (11.03)	51.48 (12.42)
*P*-value		0.543	0.086	0.342	**0.002**	0.634	0.743
Ethnicity	Dalit/Janajati	50.42 (9.28)	52.83 (10.63)	56.33 (8.68)	53.06 (11.54)	51.22 (10.34)	54.38 (10.06)
	Madeshi	51.13 (7.42)	53.54 (9.56)	55.42 (12.34)	50.28 (9.84)	54.79 (12.02)	49.64 (12.33)
	Muslim	54.23 (11.64)	52.12 (11.62)	50.12 (9.63)	51.62 (12.43)	53.11 (10.44)	52.73 (9.23)
	Brahmin/Chhetry	53.22 (10.04)	54.67 (8.45)	51.62 (11.24)	52.43 (14.56)	50.45 (11.84)	53.61 (8.86)
*P*-value		0.726	0.541	0.823	0.431	0.562	0.312
Family Type	Single	57.06 (5.66)	53.45 (6.37)	51.36 (11.41)	53.51 (8.62)	55.97 (10.36)	54.21 (8.33)
	Joint	54.42 (8.96)	56.21 (10.46)	52.13 (12.44)	57.62 (6.12)	52.63 (11.25)	54.37 (6.39)
*P*-value		0.063	0.126	0.078	0.643	0.563	0.052
Socio-economic status	Upper/Upper middle	57.42 (9.12)	53.48 (8.44)	56.44 (7.23)	59.36 (9.87)	57.56 (11.02)	56.12 (12.93)
	Middle/ lower middle	54.21 (10.48)	55.87 (10.36)	57.76 (11.14)	58.78 (14.54)	56.45 (11.07)	55.39 (9.63)
	Lower/upper lower	51.62 (8.41)	50.13 (9.36)	58.37 (9.87)	56.47 (11.47)	53.22 (7.63)	49.35 (12.67)
*P*-value		0.816	0.236	0.479	0.973	0.643	0.622
Educational status	Literate	55.62 (7.66)	58.63 (9.47)	58.78 (11.36)	53.24 (12.49)	51.33 (7.36)	49.35 (12.67)
	Illiterate	57.23 (6.83)	55.44 (7.43)	58.63 (12.36)	50.41 (9.57)	51.18 (7.72)	53.56 (8.31)
*P*-value		**0.021**	0.073	0.057	0.082	0.078	0.067
Smoking habit	Yes	53.79 (12.43)	58.72 (11.21)	53.46 (15.78)	50.44 (13.61)	52.47 (9.35)	55.87 (9.92)
	No	54.37 (11.68)	50.12 (13.47)	49.37 (9.87)	55.46 (10.47)	51.25 (11.53)	54.29 (10.41)
*P*-value		0.237	0.649	0.985	0.583	**0.038**	0.732
Alcohol consumption	Yes	58.47 (8.63)	51.06 (9.31)	54.75 (6.72)	50.08 (7.31)	55.74 (10.44)	48.35 (11.25)
	No	53.67 (12.31)	57.58 (11.04)	49.08 (12.93)	54.21 (10.47)	57.09 (9.81)	54.37 (7.74)
*P*-value		0.421	0.513	0.435	0.916	0.541	0.671
Duration of disease	< 5 years	58.42 (13.42)	59.32 (12.44)	51.76 (10.57)	57.39 (15.09)	50.07 (14.11)	57.23 (10.73)
	≥5 years	50.51 (7.83)	48.63 (9.43)	54.03 (12.09)	53.02 (11.72)	54.44 (10.66)	53.03 (9.13)
*P*-value		**0.002**	0.078	0.063	**0.001**	0.088	0.059
Presence of co-morbidity	Yes	55.96 (12.47)	50.31 (13.77)	52.77 (10.29)	54.67 (10.38)	51.38 (9.67)	54.78 (11.41)
	No	58.63 (8.33)	54.67 (9.47)	53.38 (18.34)	55.39 (15.09)	56.74 (11.38)	48.43 (12.07)
*P*-value		**0.042**	0.057	**0.034**	0.051	0.073	0.081
Presence of complication	Yes	57.38 (12.61)	55.08 (9.42)	59.02 (10.05)	49.06 (11.23)	57.04 (15.68)	50.78 (13.47)
	No	54.14 (14.33)	53.81 (9.37)	51.21 (8.53)	50.51 (9.59)	55.09 (11.14)	53.35 (10.67)
*P*-value		0.344	0.516	0.734	0.819	0.547	0.539
Family history of diabetes	Yes	55.79 (7.04)	51.72 (12.54)	55.37 (13.09)	53.48 (11.57)	51.73 (12.22)	51.43 (13.46)
	No	49.37 (9.57)	55.76 (10.13)	56.43 (10.35)	56.27 (13.47)	52.94 (12.17)	49.03 (11.63)
*P*-value		0.573	**0.042**	0.874	0.071	0.439	0.563

^a^Pearson’s correlation

## Discussion

This study assessed the dimensions in which the quality of life of the T2DM residing in Baniyani VDC was affected.

The result revealed various dimension of quality of life of the diabetic patient that is affected. Highest score of quality of life was found in social burden dimension. It indicates that diabetic patients are getting good support in the society. Domain energy and mobility with least score was also found to be affected. It shows that respondents were feeling less energy affecting their daily life. Because of diabetes they were getting problem in walking and fulfilling their daily requirements. Similarly a study from Brazil and United States found contradicting result that the dimensions sexual functioning and diabetes control was greatly affected [10, 16]. But another study [17], shows that the dimension diabetes control is more affected than sexual functioning. Although both of the results have been supported by the study conducted in Nepal [11].

This study found a negative correlation of age with domains sexual functioning and energy and mobility. It describes that sexual life of a diabetic patient is affected with increasing age. Result revels that the sexual functioning of the participant is determined by their gender. Similar result has been demonstrated by the study conducted in Iran indicating the association of gender difference with quality of life [18].

This study found that educational status also affects their sugar level. This indicates that educational level enhances the skill of diabetic patient to bring their sugar level in control. Relationship between education and quality of life was demonstrated by study conducted in Turkey [19]. Dimensions sexual functioning and diabetes control were greatly affected by number of year they were diagnosed with diabetes. Complications associated with diabetes might have impact over the sexual life and create obstacles during it management. Contrarily, another study found relationship between duration of disease with energy and mobility [11].

Having co-morbidity is associated with social burden, affecting the social relationship within the community and thus the quality of life of the diabetic patient residing in eastern Nepal. Presence of co-morbidity may affect mobility of the diabetic patient which creates barrier in involving social functions and events. Previous study conducted in Mexico and South India found similar result [20, 21]. Association between family history of diabetes and the dimension anxiety and worry was found by this study. It indicates that those diabetic patients with family history were worried about the condition affecting the quality of life. Although this study did not find any relationship between quality of life and socio-economic status, study from refugee camps of Gaza strip found strong impact of economic status of the diabetic people over the quality of life [22]. Due to increased access of health care facility, free medication and consultation might have reduced the impact of economic status in management of diabetes in eastern Nepal.

This is perhaps the first community based study conducted in Nepal using diabetes-39 questionnaire in order to assess the quality of life of diabetic patient. The study is limited to a single VDC of eastern Nepal so, we suggest further studies including large sample size for better understanding of the dimensions of quality of life of diabetic patient.

## Conclusion

This study found that with increasing age sexual life and mobility of the diabetic patient is also affected. The domain sexual functioning is difference by sex and having co-morbidity also have difference in their sexual life. Similarly, maintaining blood sugar level is affected by duration of disease and educational status of the patient. And having family history of diabetes affects the mental state of the diabetic patient. Hence it is needed to focus on screening program and diabetic education in community level in order to limit its complications and to increase the quality of life of type 2 diabetic patient.

## Data Availability

Entered data are available on request from the corresponding author.

## Abbreviations

BPKIHS: BP Koirala Institute of Health Sciences
COHESION: Community Health System Innovation
FCHVs: Female Community Health Volunteers
IRC: Institutional Review Committee
PHCC: Primary Health Care Centre
VDC: Village Development Committee
